# High-Speed Tableting of High Drug-Loaded Tablets Prepared from Fluid-Bed Granulated Isoniazid

**DOI:** 10.3390/pharmaceutics15041236

**Published:** 2023-04-13

**Authors:** Valentyn Mohylyuk, Dace Bandere

**Affiliations:** 1 Laboratory of Finished Dosage Forms, Faculty of Pharmacy, Riga Stradiņš University, LV-1007 Riga, Latvia; 2 Department of Pharmaceutical Chemistry, Faculty of Pharmacy, Riga Stradiņš University, LV-1007 Riga, Latvia

**Keywords:** isoniazid, tablets, high drug-loaded, granulation, wet granulation, fluid-bed granulation, high-speed tableting, Heckel plot, compressibility, tabletability, compactability

## Abstract

The aim of this feasibility study was to investigate the possibility of producing industrial-scale relevant, robust, high drug-loaded (90.9%, *w*/*w*) 100 mg dose immediate-release tablets of isoniazid and simultaneously meet the biowaiver requirements. With an understanding of the real-life constrictions on formulation scientists during product development for the generic industry, this study was done considering a common set of excipients and manufacturing operations, as well as paying special attention to the industrial-scale high-speed tableting process as one of the most critical manufacturing operations. The isoniazid substance was not applicable for the direct compression method. Thus, the selection of granulation method was logically justified, and it was fluid-bed granulated with an aqueous solution of Kollidon^®^ 25, mixed with excipients, and tableted with a rotary tablet press (Korsch XL 100) at 80 rpm (80% of the maximum speed) in the compaction pressure range 170–549 MPa monitoring of ejection/removal forces, tablet weight uniformity, thickness, and hardness. Adjusting the main compression force, the Heckel plot, manufacturability, tabletability, compactability, and compressibility profiles were analysed to choose the main compression force that resulted in the desirable tensile strength, friability, disintegration, and dissolution profile. The study showed that highly robust drug-loaded isoniazid tablets with biowaiver requirements compliance can be prepared with a common set of excipients and manufacturing equipment/operations incl. the industrial-scale high-speed tableting process.

## 1. Introduction

Worldwide, tuberculosis is the 13th leading cause of death and the second leading infectious killer after COVID-19 (above HIV/AIDS). Worldwide in 2021, a total of 1.6 million people died from tuberculosis, and an estimated 10.6 million people fell ill with tuberculosis including 6 million men, 3.4 million women, and 1.2 million children [1].

Isoniazid (isonicotinic acid hydrazide) is an antibiotic which is one of the key substances used in the first-line treatment or prevention of tuberculosis and in most cases is used in the form of tablets [2]. As a result, isoniazid tablets with doses of 100 and 300 mg are listed on the WHO Essential Medicines List [3].

Isoniazid has a solubility greater than 150 mg/mL in water and solution in pH range of 1.2–6.8 [2]. Polymorphism and hydrates of isoniazid were reported as well as the formation of orthorhombic crystals upon synthesis [4]. In accordance with the biopharmaceutical classification system (BCS), Isoniazid was classified on the borderline of Class I and Class III, and was recommended for the biowaiver procedure in case of fulfilling the FDA, EMEA, and WHO requirements for “very rapidly dissolving” product, which is 85% dissolution of the labelled amount of isoniazid within 15 min in pH 1.2, 4.5, and 6.8, using the basket apparatus at 75 rpm [2].

Isoniazid tablets are prescribed to patients from different age groups, including paediatric and geriatric population subgroups, as well as people with swallowing difficulties. Thus, the size of tablets is especially important to avoid complicated tablet swallowing as a reason for skipped dose or discontinued treatment and to achieve desirable patient compliance [5,6]. From the regulatory point of view, the size, shape, and other physical attributes of tablets are dictated by original/reference product [6]. The originator products for isoniazid no longer exist, however, the medicinal product Isozid (Fatol Arzneimittel GmbH, Schiffweiler, Germany) with a quite long and well-established marketing history is very often used as a reference product [7,8].

The average mass of an Isozid 100 mg dose tablet is 110 mg (incl. hypromellose, copovidone, crospovidone, silica dioxide, microcrystalline cellulose, magnesium stearate, macrogol 6000, and talc.), meaning that the tablet with 90.9% of isoniazid content can be classified as a high drug-loaded tablet. Despite the recently proposed approaches for formulation of cocrystals and eutectics [9,10,11], 3D-printed tablets with personalised dosing and drug release [12], and child-friendly dispersible tablet [13], the manufacturing of conventional 100 mg dose isoniazid tablets with immediate release is still favourable due to an already accumulated wealth of medical knowledge [14] and demand by contemporary healthcare system in many countries [3].

The current pharmaceutical/generic industry has ubiquitously available equipment, such as mixers, granulators-dryers, and tableting machines as well as knowledge, and existing facilities to produce conventional tablets on an industrial scale. In turn, these allow for the organization of the mass production of generic products in the shortest terms and with minimal investment in production sites, in safety/efficiency proof, and minimal efforts in regulatory approval.

In real life, during the development of generic products, formulation scientists are usually limited by many factors such as available production sites, available processing equipment, and the existing list of excipients. These factors often influence the decision tree and final formulation. Additionally, the formulation and technological process should meet not only the expectations in quality but performance indicators too. One of the most critical indicators is the speed of industrial-scale tableting process. It should be noted, the smaller the weight of the tablet, the longer the tableting process. Thus, the tableting process should be as fast as possible while achieving of desirable pharmaco-technological and biopharmaceutical properties of tablets.

In such a high drug-loaded formulation, the properties of isoniazid have a huge impact on the processability/manufacturability and the amount of excipient needed to achieve desirable uniformity, flowability, industrial-scale high-speed tableting, and biowaiver requirements limited to 9.1% [15]. A special challenge is achieving the desired mechanical properties of highly drug-loaded tablets when using high-speed tableting because their mechanical properties are influenced by the properties of the raw drug substance.

This work was done as a feasibility study aimed to investigate the possibility of producing high drug-loaded (90.9%, *w*/*w*) 100 mg dose immediate-release tablets of isoniazid with desirable properties, utilizing a widely used common set of excipients and a common manufacturing operation, including fluid-bed wet granulation with aqueous binder solution, and industrial-scale high-speed tableting. The rationale of excipient choice and their concentrations were not proved here with experimental results as they are well-known approaches for the professional pharmaceutical community. Instead, the main focus was given to the industrial-scale high-speed tableting process (as one of the most critical manufacturing operations); specifically, to the Heckel plot, manufacturability profile, and the compaction triangle: tabletability, compactability, and compressibility profiles. The following task was to use the obtained tableting process-related results to determine the desirable tablet compression force at a given tableting speed and to test the fitting of dissolution results to the proposed biowaiver requirements.

## 2. Materials and Methods

Isoniazid (INZ; Vaishaly Pharmaceuticals, Mumbai, India; batch # T2005 195/INH) was used as the active pharmaceutical ingredient, povidone (Kollidon^®^ 25; BASF SE, Ludwigshafen, Germany) was used as binder for wet granulation, modified waxy maize food starch (Resistamyl^®^ 347; Tate & Lyle PLC, Koog aan de Zaan, The Netherlands) was used as diluent-disintegrant, crospovidone (Polyplasdone^TM^ XL-10; Ashland Specialty Ingredients, Wilmington, DE, USA) was used as superdisintegrant, while magnesium stearate (Sudeep Pharma Pvt. Ltd., Nandesari, Vadodara, India) was used as lubricant. Isozid^®^ 100 mg tablets (Fatol Arzneimittel GmbH, Schiffweiler, Germany) were used as reference. Chemicals used for preparation of dissolution media were of Pharmacopoeia grade and used as received.

### 2.1. Moisture Content

The moisture content in approx. 500 mg of sample was determined using a moisture analyser (MB45; Ohaus Corp., Greifensee, Switzerland) at 105 °C isothermal heating until constant mass (*n* = 3).

### 2.2. Differential Scanning Calorimetry (DSC)

To investigate the thermal properties of raw isoniazid and crushed tablets, a heat-flux DSC (DSC Q20; TA Instruments, New Castle, DE, USA) was conducted to characterize thermal behaviour. For measurement, samples were weighed (5–8 mg) in aluminium DSC pans and heated from 30 °C to 220 °C at 50 °C/min with a continuous purge of nitrogen gas at 50 mL/min (*n* = 3). Before analysis, the DSC equipment was calibrated with high purity indium.

### 2.3. Determination of Particle Size Distribution by Sieving Method

Powder material (50 g), sieves with a mesh size of 0.05, 0.1, 0.25, 0.5, 0.7, 1.0, and 1.25 mm and vibratory sieve shaker (AS 200 Basic; Retsch GmbH, Haan, Germany) at 1 mm amplitude for 5 min were used for the determination of particle size distribution (*n* = 3). *D*_10_, *D*_50_, and *D*_90_ were graphically extracted from the cumulative weight fraction which was generated from the raw data. The Span was used as an indicator of particle size distribution and calculated using the following equation:

$$\mathrm{Span}=\frac{D_{90}-D_{10}}{D_{50}}$$

### 2.4. Bulk and Tapped Density Testing

To investigate the bulk and tapped density of the powder mixture before and after the granulation, as well as the tableting mixture, a Tapped Density Tester (PT-TD300; Pharma Test Apparatebau AG, Hainburg, Germany) was used. The bulk and tapped density of samples were investigated using 100 g of powder material and graduated volumetric cylinder. The volume of material was visually recorded before tapping (bulk density, *ρ_bulk_*), and after 10, 500, and 1250 taps (tapped density, *ρ_tapped_*). All measurements were made in triplicate (*n* = 3). Bulk and tapped densities as well as Hausner ratio (*HR*) were calculated using the following equations [16,17]:

$$\rho_{bulk}=\frac{\mathrm{Powder}\;\mathrm{mass}}{\mathrm{Initial}\;\mathrm{volume}}$$

$$\rho_{tapped}=\frac{\mathrm{Powder}\;\mathrm{mass}}{\mathrm{Volume}\;\mathrm{after}\;1250\;\mathrm{taps}}$$

$$HR=\frac{\rho_{tapped}}{\rho_{bulk}}$$

### 2.5. Mass Flow Rate Determination with Gravitational Funnel Method

A stainless-steel frustum cone funnel was fixed in a strictly vertical position (flowability tester; Pharma Test Apparatebau AG, Hainburg, Germany). The samples were weighed as 100 g and introduced carefully into the funnel with a 10 mm diameter orifice. During filling, the bottom opening of the funnel was closed. Once the orifice was opened, the time taken for the 100 g of powder within the funnel to flow out was measured (*n* = 6). The mass flow rate was calculated as mass per time, expressed in g/s [16].

### 2.6. Fluid-Bed Wet Granulation

**Figure 1:**
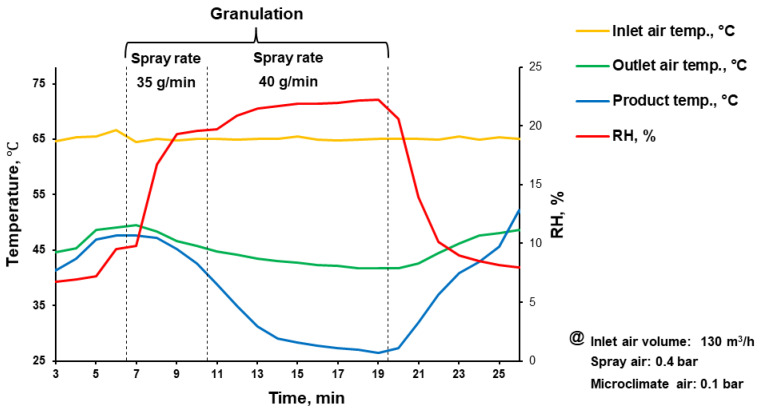
Actual parameters of fluid-bed wet granulation process.

Before granulation, the isoniazid substance was milled with a hammer mill (Bohle Turbo Mill; L.B. Bohle Maschinen und Verfahren GmbH, Ennigerloh, Germany) at 4000 rpm. For wet granulation, a pilot-scale fluid-bed granulator (Unilab 5-DJ, Hüttlin GmbH/Bosch, Schopfheim, Germany) was used, equipped with an air distribution disc (Diskjet) with two inbuilt three-component spray nozzles (the internal diameter of the binder liquid supplying nozzle was 1.2 mm) and cloth filters [18]. Milled isoniazid substance was granulated with 15% (*w*/*w*) Kollidon^®^ 25 solution in deionized water (Table 1, Figure 1). The discharged granulated material was passed through the sieve with a 1.5 mm mesh size.

### 2.7. Preparation of Tablets

Before mixing, magnesium stearate Resistamyl^®^ and Polyplasdone^TM^ XL-10 were passed through a sieve with a 0.5 mm mesh. Granulated material, Resistamyl^®^, and Polyplasdone^TM^ XL-10 in required proportions (Table 1), were mixed in a Turbula mixer for 10 min. Then, magnesium stearate was added, and the premix was additionally mixed for 5 min. To obtain 100 mg dose flat-cylindrical tablets (diameter of 6 mm) with a weight of 110 mg (Table 1), the round flat-faced punch-die sets, the rotary tablet press with 13 punch stations, and forced powder filler were utilized (model XL 100; Korsch AG, Berlin, Germany) at a constant: tableting speed of 62,400 tab/h (turret speed of 80 rpm); feed frame forced die filling speed (with the rectangular straight paddle geometry) of 40 rpm; lower punch position during the filling process of 5.7 mm; and precompression force of 0.40 ± 0.03 kN [19,20]. While the maximum recommended compression force for this punch type was 19 kN, the compaction force was varied between 4.8 and 15.5 kN. The tableting force, ejection force, and tablet removal force were registered by inbuild detectors.

### 2.8. Dwell Time Calculation

The dwell time (in milliseconds) was calculated using the following equation [21,22]:

$$T_{dwell}=\frac{D_{hf}\times 60\times 1000}{\pi \times V_{turret}\times D_{turret}}=\left[\;\frac{\mathrm{mm}}{\mathrm{rpm}\times \mathrm{mm}}=\mathrm{ms}\right]$$

where: *D_hf_* and *D_turret_* are the diameters of the punch had flat and turret; *V_turret_* is the velocity of the turret.

### 2.9. Calculation Tablets’ Tensile Strength

The tablet hardness (breaking force; *F*) was measured by a hardness tester (TBH 20; Erweka, Hessen, Germany) within 12 h after the compaction. The tablet thickness (*t*) and diameter (*d*) were measured (*n* = 10) with digital calliper (Duratool, Leeds, UK) with a resolution of 0.01 mm. Tensile strength (*τ*, MPa) was calculated by employing the following equation [23]:

$$\tau =\frac{2\;F}{\pi \;d\;t}$$

### 2.10. Calculation of Tablets’ Pore and Solid Fraction

The theoretical true density of tablet composition was calculated based on the true density (*ρ_t_*) of isoniazid [24] and excipients [25] and their shares (*x*, *w*/*w*) using the additive methodology and the following equation [26]:

$$\rho_{t}=\left(\rho_{INZ}\cdot x_{INZ}\right)+\left(\rho_{exc.1}\cdot x_{exc.1}\right)+\ldots \left(\rho_{exc.i}\cdot x_{exc.i}\right)$$

The relative volumes and densities of the tablets were determined after ejection from the die. Apparent density (*ρ_a_*) of tablets was calculated as the ratio of tablet weight (*w_tab_*) and volume of cylinder using the following equation:

$$\rho_{a}=\frac{w_{tab}}{\pi {\left(\frac{d}{2}\right)}^{2}t}$$

Pore (*ε*) and solid fraction (*SF*) of tablet was calculated using the following equations [27]:

$$SF=\frac{\rho_{a}}{\rho_{t}}=\left(1-\varepsilon \right)$$

$$\varepsilon =1-\frac{\rho_{a}}{\rho_{t}}=\left(1-SF\right)$$

### 2.11. Heckel Plot Construction

The relative density (*ln*(1/*ε*)) and compaction pressure (*P*, MPa) data were plotted in accordance with Heckel equation:

$$\ln\left(1/\varepsilon \right)=K\cdot P+\ln\left(1/\varepsilon_{0}\right)=K\cdot P+A$$

where: *K* is the slope of the linear region (the proportionality constant), and *ln*(1/*ε*_0_) is a constant, A, that represents the degree of packing (at pore fraction *ε*_0_) achieved at low pressure because of the rearrangement process before an appreciable amount of interparticle bonding takes place [28].

The yield pressure (*P_y_*, MPa) was calculated in accordance with Hersey and Rees by the equation [29]:

$$P_{y}=\frac{1}{K}$$

### 2.12. Weight Uniformity

The tablet press was preset to prepare 110 mg tablets. Twenty tablets were individually weighed, and the average (Av.) weight and weight relative standard deviation (R.S.D.) were calculated. Then, the individual tablet weights were compared to the mean and the difference (%) was calculated. In our case (being in the range of 80–250 mg), to meet European Pharmacopoeia requirements (General Monograph 2.9.5. “Uniformity of mass of single-dose dosage forms”), not more than two of the individual weights deviated from the average weight by more than 7.5% and none deviate by more than 15%.

### 2.13. Determination of Tablet Friability

The friability test was conducted using an automatic drum tablet friability instrument (PTF 20E; Pharma Test Apparatebau AG, Hainburg, Germany) at a fixed rotation speed and test duration (25 rpm for 4 min) in accordance with European Pharmacopoeia General Monograph 2.9.7. “Friability of Uncoated Tablets”. To measure the friability, 20 tablets (*n* = 20) were dedusted, weighed, and loaded into the testing drum. After the friability test, tablets were dedusted, weighed, and the friability (weight loss) of tablets was calculated and expressed as a percentage of the initial weight (%) (*w*/*w*) [30]. The friability test was repeated five times (*n* = 5) for every tableting condition.

### 2.14. Determination of Tablets Disintegration Time

Disintegration time was determined with a disintegration tester (Pharma Test Apparatebau AG, Hainburg, Germany) according to the European Pharmacopoeia General Monograph 2.9.1. “Disintegration of tablets and capsules” [31], in 900 mL demineralized water at 37 ± 0.5 °C. Six tablets (*n* = 6) were tested for each tableting condition of interest.

### 2.15. Testing the Isoniazid Release from Tablets

The release was tested using the USP-I (basket) apparatus at 37 °C in 900 mL of 0.1 N HCl solution (pH 1.2), acetate buffer (pH 4.5), or phosphate buffer solution (pH 6.8) at 75 rpm. At the predetermined time points, the aliquot (2 mL) was withdrawn and filtered. The withdrawn aliquot volume was replaced with fresh dissolution medium. To quantify isoniazid, the samples were diluted 5 times with deionized water to fit a concentration below 50 μg/mL and the isocratic HPLC method with an automatic injection of 20 μL was used. C18 column (5 μm, 4.6 × 100 mm) at 30 °C and the mixture of 20 mM NaH_2_PO_4_ (which was adjusted to pH = 6 with phosphoric acid) and methanol (85:15) as mobile phase at a flow rate of 1 mL/min was used. After injection, the absorbance was recorded with a UV detector at λ_max_ 262 nm and calculated based on the isoniazid peak area and calibration curve (Y = 3.625 × 10^4^·X + 0.25 × 10^4^, R^2^ = 0.9994) [32].

## 3. Results

**Figure 2:**
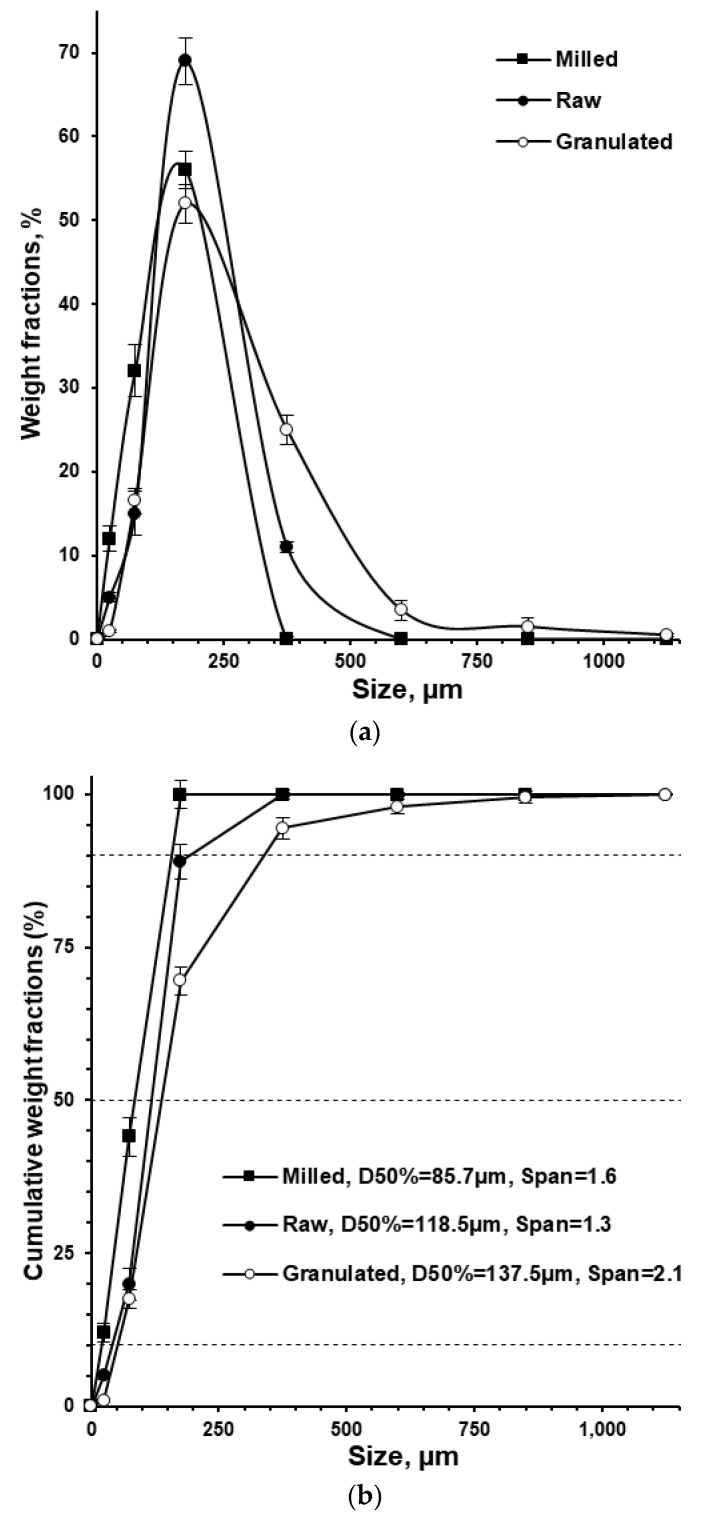
Particle size distribution (**a**) and cumulative weight fractions (**b**) of raw, milled, and granulated isoniazid (*n* = 3).

**Figure 3:**
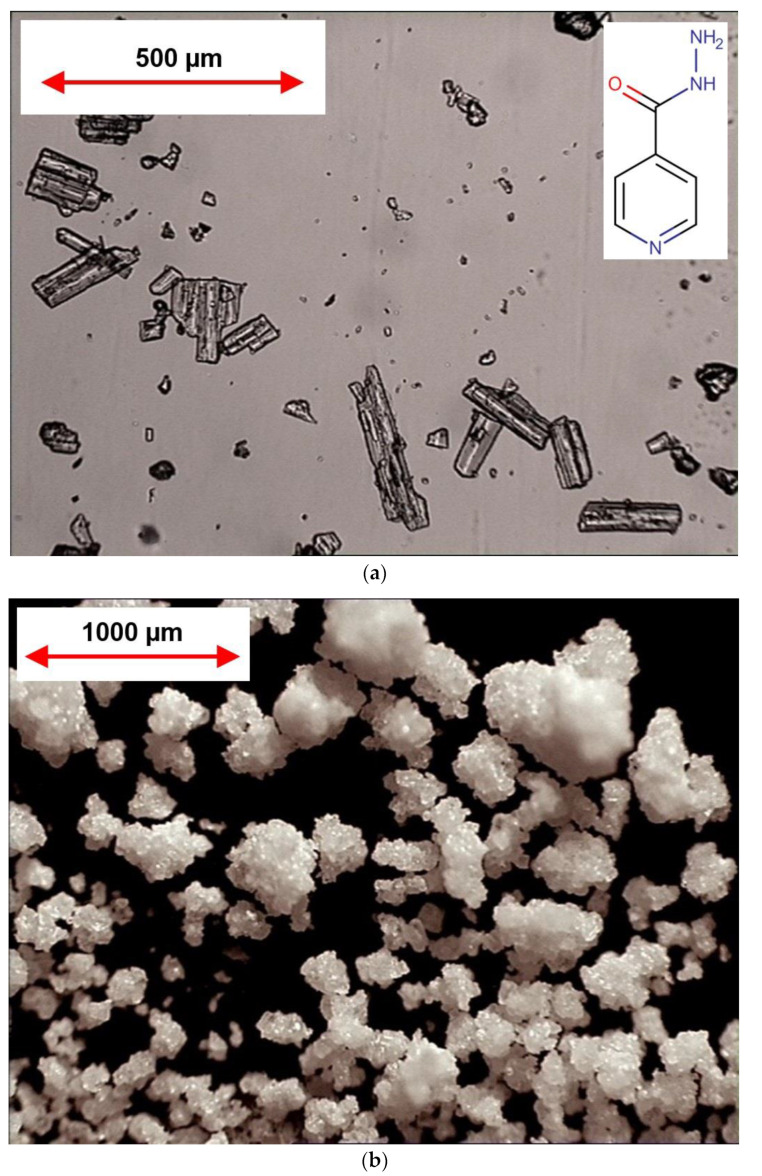
Light microscopy images of raw ((**a**); transmitted light/bottom highlighting) and granulated ((**b**); reflected light/top highlighting) isoniazid.

For successful granulation process, meaning achieving homogeneous distribution of binding excipient (povidone) and desirable mechanical properties, the raw isoniazid substance was milled before the granulation. After milling, the particle size (*D_50_*) of raw isoniazid was decreased from 118.5 to 85.7 µm while the span of the particle size distribution was increased from 1.3 to 1.6, respectively (Figure 2 and Figure 3a).

Kollidon^®^ 25 (povidone) was used as a wet granulation binder at a concentration of 3.1% (*w*/*w*; based on the dry weight of granulate) and 3.0% (*w*/*w*; based on tablet weight) while the usual range of povidone as tablet binder is up to 5% (*w*/*w*) [25]. The Kollidon^®^ 25 concentration in aqueous solution intended for granulation was 15% (*w*/*w*).

**Figure 4:**
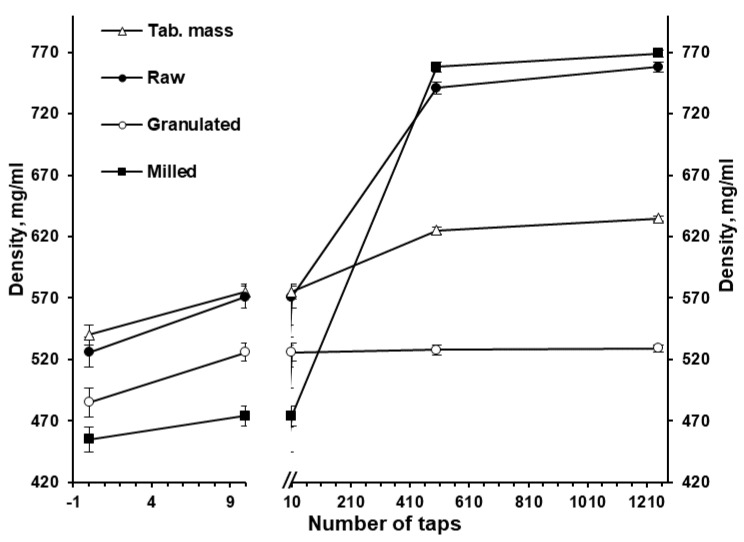
The effect of tapping on the apparent density of raw, milled, and granulated isoniazid as well as mixture for tableting (*n* = 3).

**Figure 5:**
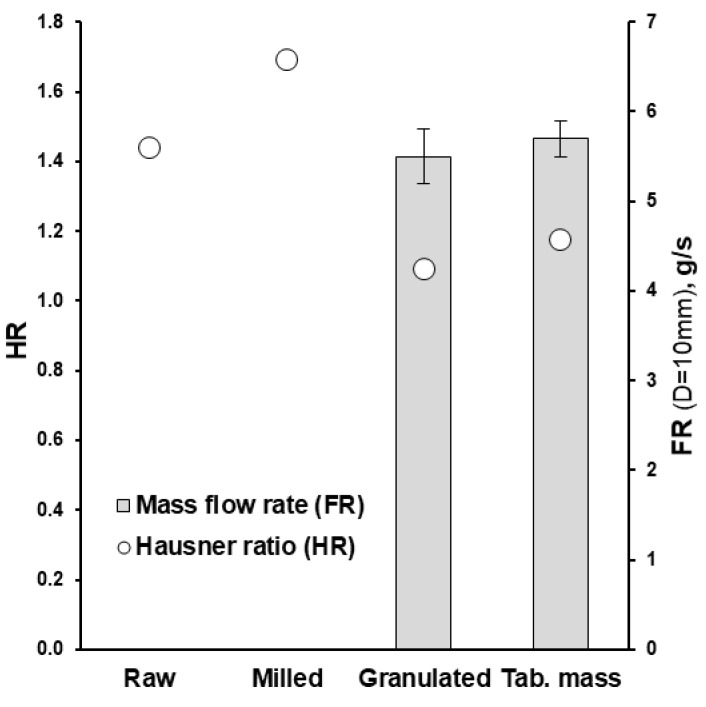
The effect of fluid-bed wet granulation on the mass flow rate (FR; *n* = 3) and Hausner ratio (HR) of granules and the tableting mixture.

The granulation (Figure 1) drastically changed the powders’ appearance (Figure 3a vs. Figure 3b), particle size, apparent density and densification kinetics, mass flow rate, and Hausner ratio. The size of granules (*D_50_*) comprised 137.5 µm with the span of particle size distribution of 2.1 (Figure 2). Granulated material almost did not contain fine particles below 50 µm (0.9 ± 0.2% based on total granules sample) and upon tapping the apparent density was lower than the milled or raw substance. While the bulk density of milled and granulated isoniazid was close to each other (455 ± 10 and 485 ± 12 mg/mL) the tapped density of milled isoniazid was much higher (769 ± 3 and 529 ± 3 mg/mL, respectively) (Figure 4). The less efficient packaging and lower tapped density of granulated isoniazid can be attributed to a porous granule structure and the granule shape. While the raw and milled isoniazid were non-flowable, the granulated one demonstrated the mass flow rate through the funnel with 10 mm orifice at the level of 5.5 ± 0.3 g/s (Figure 5).

**Figure 6:**
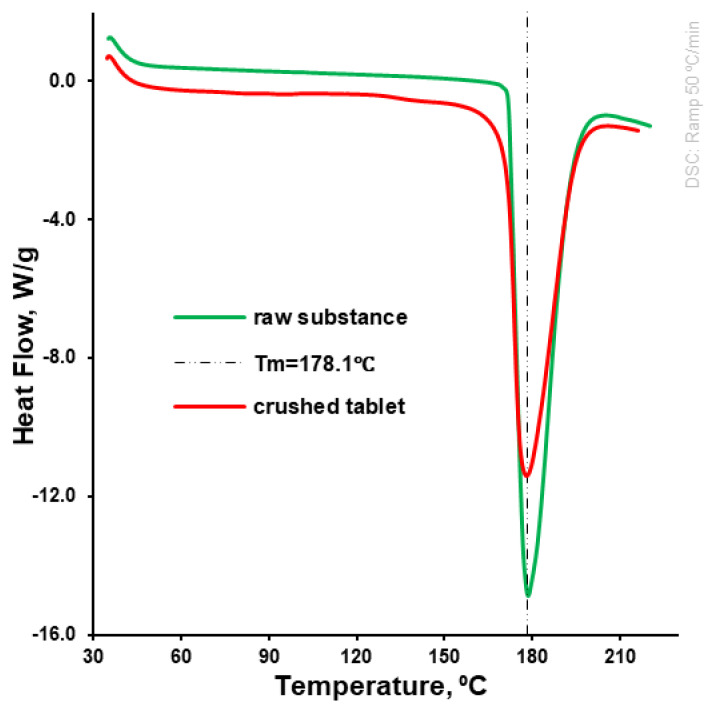
DSC profile of raw isoniazid and crushed tablet.

The moisture content in raw and milled isoniazid substance was determined at the level of 0.03%, while in the granulated isoniazid the moisture content was 0.26% (*w*/*w*). Thus, the formation of hydrates during the fluid-bed wet granulation was not expected. The milling, fluid-bed wet granulation, and tableting of isoniazid did not change its solid state. This was confirmed by a comparison of DSC profiles of raw vs. milled, granulated, and tableted substances—crushed tablet (Table 2, Figure 6). Specifically, the melting point remained unchanged (around 178 °C), while the melting endotherm of crushed isoniazid tablet was slightly lower (228.6 ± 2.2 vs. 204.3 ± 5.6 J/g) and almost proportional to the isoniazid content in DSC samples (Figure 6). The values of melting point and melting endotherm of raw isoniazid were very close to values determined in the independent research [10].

To achieve desirable disintegration and dissolution properties, and to standardize tablet weight, the granules were mixed with the superdisintegrant Polyplasdone^TM^ XL-10 (crospovidone) and the diluent-disintegrant Resistamyl^®^ 347 (modified starch). Polyplasdone^TM^ XL-10 and Resistamyl^®^ 347 were used in the concentration of 2.3% and 3.3%, respectively, while the usual range is 2–5% and more than 5% (*w*/*w*; based on tablet weight), respectively. To reduce the friction after compression upon tablet ejection from the die, in the tableting composition mixture, magnesium stearate was introduced as a lubricant at a concentration of 0.5% (*w/w*; based on tablet weight) [25].

Because of the relatively small excipients’ particle size and consequent filling of the voids with small particles in the powder mixture, and also due to the using magnesium stearate as a lubricant and reduction of particle-to-particle friction, the bulk and tapped density of tableting mass were higher than for granulated isoniazid (540 vs. 485 and 635 vs. 529 mg/mL, respectively; Figure 4). Alternatively, the flowability of granules and tableting mass through the gravitational funnel with a 10 mm orifice were mostly comparable (Figure 5). The values of Hausner ratio were in agreement with the flowability results (Figure 5). During the tableting process, no rat-holing, arching, or other powder flow-related problems were observed in the hopper of the rotary tablet press.

**Figure 7:**
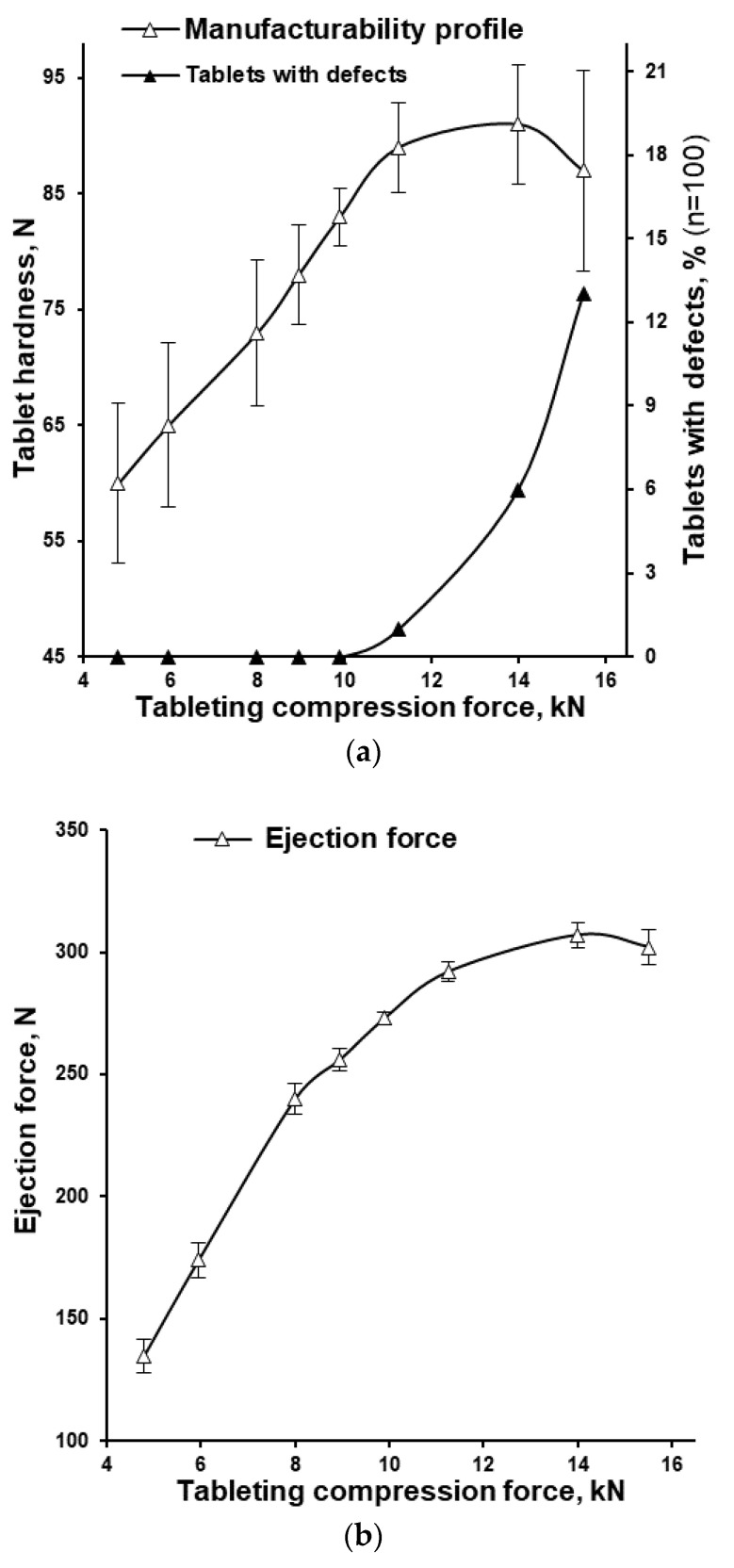
Effect of tableting compression force on: (**a**) the tablet hardness (Manufacturability profile; *n* = 20) and percent of tablets with defects after friability test (*n* = 100); (**b**) the tablet ejection force from the die (*n* = 100).

The obtained tableting mass was tableted using the rotary tablet press Korsch XL 100. During the adjustment and for a quick indicative assessment, the initial tablets were prepared at 8 rpm (193 ms dwell time) in the range of tableting forces up to 18 kN and the highest possible tablet hardness of 194 ± 5 N (Av. ± S.D., *n* = 10) was achieved. All following tableting experiments were performed at 80 rpm (19 ms dwell time; 62,400 tab/h) at different tableting compaction force in the range of 4.8–15.5 kN and during tableting process, the tablets demonstrated an acceptable weight uniformity with tablet weight R.S.D. in the range of 1.6–5.0 (Table 3). On the **manufacturability profile** (tablet hardness, N vs. tableting compaction force, kN; Figure 7a), the increase of compaction force from 4.8 to 11.3 kN increased the tablet hardness in an almost linear manner. The compaction force of 11.3 kN resulted in a tablet hardness of 89 N but following an increase in the compaction force, the tablet hardness did not improve (overlapping of error bars representing S.D.) but only served to increase the percent of tablets with defects after the friability test (Figure 7a). Specifically, at a compaction force of 11.3 kN, only 1% of tablets with defects were observed, while at 14.0 and 15.5 kN, 6 and 13% of tablets with defects were observed, respectively. At the same time, the increase of compaction force from 4.8 to 14.0 kN demonstrated the ascending profile of tablet ejection force with a peak value of 307 ± 5.5 N followed by slight decrease of ejection force of 302 ± 7.0 N at compaction force of 15.5 kN (Figure 7b). In the mentioned range of compaction forces (4.8–15.5 kN), the tablet removal force from the surface of the lower punch after the ejection from the tablet die was in the range of 0.5–09 N. The tablet ejection and tablet removal force were in the acceptable range across the full range of tested tableting compression forces.

**Figure 8:**
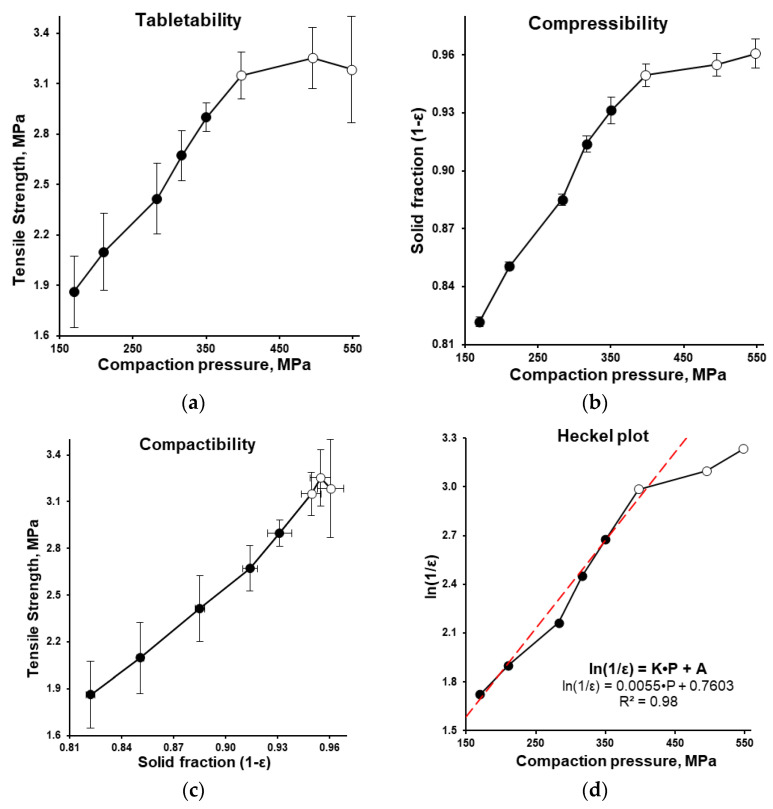
Tabletability (**a**), compressibility (**b**) and compactability (**c**) profiles as well as Heckel plot (**d**) (*n* = 10). Empty markers depicted conditions that resulted in the appearance of defective tablets after the friability test.

At the following stage, based on the measured and calculated tableting characteristics (Table 3), tabletability, compactability, and compressibility profiles (the **compaction triangle**) were built. The same as the manufacturability profile, in accordance with the **tabletability profile** (tensile strength, MPa vs. tableting compaction pressure, MPa; Figure 8a), the increase of tableting compaction pressure from 169.9 to 398.1 MPa increased the average tensile strength in an almost linear manner. At a compaction pressure of 398.1 MPa and higher (specifically, at 495.4 and 548.5 MPa) the tensile strength of tablets was almost the same (3.25 and 3.18 MPa, respectively) and the percent of tablets with defects (after the friability test) increased along with the increase of compaction pressure (Figure 7a).

Based on the apparent density (Figure 4), the solid fraction of bulk and tapped tableting mass was 0.383 and 0.450, respectively. The **compressibility profile** (solid fraction vs. compaction pressure, MPa; Figure 8b) showed the average solid fraction increase from 0.822 to 0.950 (almost in the linear manner) along with the tableting compaction pressure increase from 169.9 to 398.1 MPa, respectively. At compaction pressure of 398.1 MPa and higher (specifically, at 495.4 and 548.5 MPa) the solid fraction increase was slowed from 0.950 ± 0.006 to 0.955 ± 0.006 and 0.961 ± 0.008, respectively (Figure 8b).

The **compactability profile** (tensile strength, MPa vs. solid fraction; Figure 8c) showed the increase of the average tensile strength from 1.86 to 3.25 MPa (almost in the linear manner) along with the average solid fraction increase from 0.822 to 0.955, respectively. The difference between the solid fraction and tensile strength of tablets obtained at 11.3–15.5 kN of compaction force (398.1–548.5 MPa of compaction pressure, respectively) depicted as the three upper points of the profile on the graph was very small (Figure 8c).

The **Heckel plot** (relative density, *ln*(1/*ε*) vs. tableting compaction pressure, MPa; Figure 8d) demonstrated an approximately linear dependence (R^2^ = 0.98) in the compaction pressure range of 169.9–398.1 MPa (the compaction force range of 4.8–11.3 kN, respectively). In accordance with the Heckel equation, the proportionality constant *K* and yield pressure (*P_y_*) were equal to 0.0055 and 181.8 MPa, respectively, and the constant A was equal to 0.7603 that corresponded to pore fraction *ε_0_* of 0.468 (and solid fraction of 0.532).

**Figure 9:**
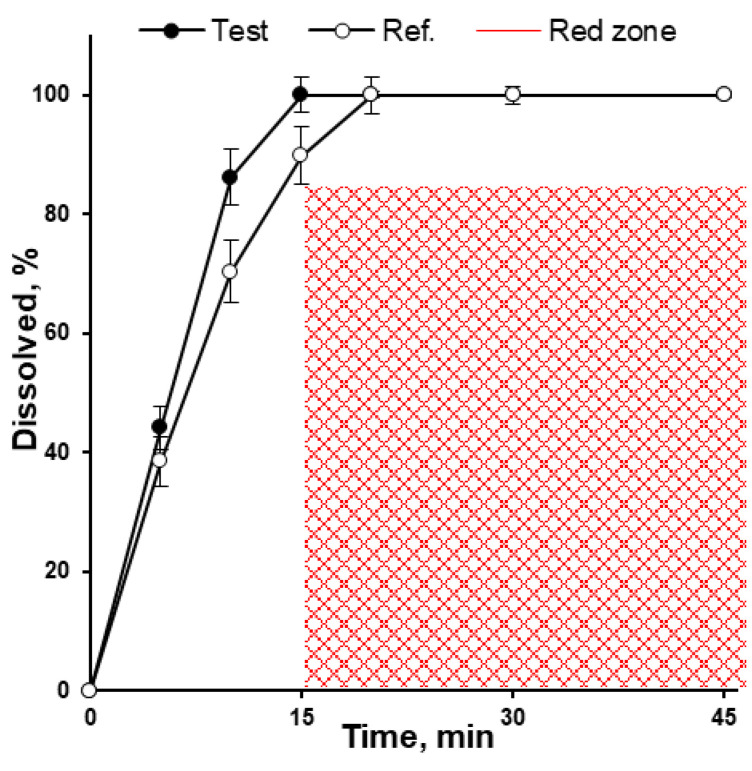
Isoniazid tablets drug release testing in 900 mL volume of pH 1.2 at 75 rpm (*n* = 6). The “red zone” is showing the area beyond of biowaiver specification for the dissolution test [2].

The unchanged solid state of isoniazid after the manufacturing process (incl. tableting) was confirmed by DSC (Figure 6). The tablets obtained at a compaction force of 9.0 kN demonstrated a disintegration time of 4.0 ± 0.5 min, as compared to 5.0 ± 1.0 min for the reference product. The dissolution rate for test and reference tablets was higher than 85% per 15 min in 900 mL dissolution media at a pH of 1.2, while the dissolution rate for test tablets was significantly higher (*p* = 0.01%) when compared with reference tablets (99.9 ± 2.9% vs. 89.8 ± 4.8%, respectively; Figure 9). In the dissolution media, with pH of 4.5 and 6.8, test tablets showed a dissolution rate higher than 85% per 15 min as well (data was not presented), and thus met the proposed biowaiver requirements.

## 4. Discussion

Isoniazid is a caking, non-flowable substance and not suitable for a direct compression method (Figure 5). The crystal structure of the molecular solid is responsible for its mechanical properties. In the brittle zigzag molecular crystal lattice of isoniazid, each isoniazid molecule forms synthons with three adjacent isoniazid molecules with pyridine···HNH− and two azide···azide functionalities having −HN···H2N− interactions. Thus, isoniazid crystals are accompanied by low compressibility and high yield pressure (337 MPa) [10]. Granulation (wet or dry/roll-compaction granulation), as a manufacturing operation, is usually used to improve flowability and compressibility of powder, as well as to avoid the segregation of mixture.

Granulation is a purposeful process to agglomerate particles due to the formation of solid bridges between them. For the formation of solid bridges, the powdered binder particles (roll-compaction) or binder solution (wet granulation) is used. As a result of bridges’ formation by wet granulation, at the contact point, the initial shape of the particle surface is changing and a new solid with specific mechanical properties is formed because of dissolving/melting and recrystallisation [33].

Due to the dissolving of the binder in a solvent, the efficiency of binder distribution on the surface of the drug particles and the changes in the drug particles’ surface is more pronounced compared with roll-compaction. Consequently, the required amount of binder to change the ability of drug powder to be applicable for tableting is lower (usually up to 2–6% *w*/*w* on a dry basis) if wet granulation is used [34,35]. At the same time, roller-compaction granulation usually requires 15% or more [36,37].

The authors assume that mentioned drug substance properties in the “Manufacturing Classification System” in order to meet the requirements of the high-shear wet granulation process are applied for fluid-bed wet granulation as well. So, in terms of melting point, wetting ability, and dose number, the isoniazid substance demonstrated the desirable properties to be considered for fluid-bed wet granulation [15].

Smaller particles are more easily aggregated into granules, facilitating the granulation process. Thus, the raw substance was preliminary milled to reduce the particle size up to *D*_50_ of 85.7 µm (Figure 2) by breaking a strong directional 3D H-bonded network of isoniazid crystals [10].

The particle size of powdered substance and the way of granulation are the factors affecting the surface area and the distribution of binding excipient on the surface of substance. In this study, we used the fluid-bed granulation with an aqueous solution of the binder. The use of organic solvents implies additional requirements for equipment and production facilities. This consequently increases the price and investment barrier and decreases the number of production sites where the technology can be implemented.

Fluid-bed granulation is a contemporary way of obtaining the granulated material by homogeneous distribution of binder on the surface of substance particles upon the spraying of binder solution [38]. Fluid-bed granulation technology has some advantages in comparison with the high-shear wet granulation of highly soluble substances such as isoniazid. Due to the simultaneous continuous wetting and drying during the fluid-bed granulation process [39], the risk of micro- and macro-overwetting and consequently unreliable granulation endpoint during the high-shear wet-granulation process can be reduced [40,41].

The used amount of binder should be enough to achieve the desirable granules and tableting mass properties as well as to satisfy a tablet’s mechanical properties, disintegration, and dissolution [35]. In this investigation, the granules were obtained at the Kollidon^®^ 25 binder level of 3.1 and 3.0% based on the granulated material and tablet weight, respectively (Table 1). The formulation used (Table 1), as well as milling, granulation (Figure 1), and mixing processes allowed for the improvement of the powder properties of the granulated material and resultant tableting mass (Figure 2, Figure 3, Figure 4 and Figure 5) without the changing of isoniazid’s solid state (Figure 6).

So, the given granulated material and the resulted tableting mass with respective properties was used to investigate the effect of tableting compression force on the properties of tablets using an industrial-scale high-speed tableting with rotary tablet press Korsch XL 100. Specifically, the tablet press was equipped with 13 punch stations and the tableting process was conducted at 80 rpm (80% of maximum speed), which is equivalent to 62,400 tab/h and dwell time of 19 ms. Throughout the tableting process, no “rat holing” or “bridging” was observed in the gravitational hoper and concurrently, the acceptable weight uniformity with R.S.D. tablet weight in the range of 1.6–5.0 was obtained (Table 3), thus confirming the suitability of the rheological powder properties.

The Heckel plot represents the degree of densification as a function of applied pressure. The linear region of Heckel plot is usually explained by plastic deformation of particles in the tablet [28]. In our case, the near linear relation (R^2^ = 0.98) in the compaction pressure range of 169.9–398.1 MPa was predetermined with the tableting compression forces of 4.8–11.3 kN (Figure 8d, Table 3). The manufacturability profile, compressibility, tabletability, and compactability profiles demonstrated the approximately linear dependency in the same range (Figure 7a and Figure 8). The highest tablet strength was obtained at a dwell time of 193 ms (8 rpm) and was approximately two times higher than at 19 ms (80 rpm) and can be also be explained with the typical time-dependent nature of plastic deformation [42,43].

After exceeding the yield pressure when the shear strength between the particles is less than the breaking strength (tensile strength of isoniazid crystals), the particles undergo viscous flow and plastic deformation as the predominant mechanism [43,44]. So, in this compaction pressure range (169.9–398.1 MPa), we can explain the increase of tensile strength of tablets along with the compaction pressure increase due to a number of reasons: the rearrangement of particles, the decrease in porosity, the increase in solid fraction, the increase of interparticle friction, breakage of weak granules’ solid bridges, plastic deformation due to the polymer binder, the consequent increase in the contact surface and contact pressure between povidone-granulated particles, and as a result formation of new compression-caused interparticle bonding (such as solid bridges and interlocking) between them.

The following analysis of the Heckel plot indicated the presence of brittle deformation at compaction pressures higher than 398.1 MPa. The value of the apparent yield pressure is indicative of the powder resistance toward its storage of plastic and/or elastic deformation. Under these high compaction pressures, the shear strength between the isoniazid particles is greater than the crystals’ tensile strength; the larger crystals are crushed into smaller particles. As a result of this, at this compaction pressure range, the brittle fracture can be considered the predominant mechanism [43,45,46]. During the brittle deformation, the formation of a new isoniazid crystal surface can be expected, which was not covered with plastic polymer binder during the granulation process. So, the tensile strength (the value reflected by the separation/fragmentation of the tablet under a load of stress) can be attributed to the less efficient interparticle bonding.

The **tabletability profile** (tensile strength, MPa vs. tableting compaction pressure, MPa; Figure 8a) showed almost the same data as manufacturability profile (tablet hardness, N vs. tableting compaction force, kN; Figure 7a). But the last one (which does not depend on the tablet size and tablet geometry) is simplifying the comparison of data from different investigations. Tabletability describes the effectiveness of the applied compaction pressure to form the tablet with a specific tensile strength and the interplay of interparticle bonding area and bonding strength [27]. Both the manufacturability profile and number of tablets with defects after the friability test showed no reason to increase the tableting compression forces higher than 11.3 and 9.9 kN, respectively (Figure 7a). This meant that from practical point of view, the tableting compression forces should be lower than 9.9 kN.

The **compatibility profile** (tensile strength, MPa vs. solid fraction; Figure 8c) is aimed to show the ability of the powder to build a sufficient tensile strength as a function of densification. Along with the compression pressure increase, the compressibility profile showed an increase in the interparticle bonding area and illustrated the increase in the interparticle bonding strength. For comparison, in accordance with the reference, the ideal material requires a compression pressure in the range of 20–125 MPa to get a solid fraction of 0.85 [15], while in our experiment the obtained tableting mass required 210.5 MPa. To be robust enough to withstand further processing including packaging, transportation, and patient handling, the tablet should have a tensile strength of more than 1.7 MPa at solid fraction lower than 0.9 [15], while in our experiment, it existed in the solid fraction range of 0.82–0.91 and the tensile strength of tablet prepared by high-speed tableting was in the range of 1.86–2.67 MPa (Table 3).

Taking into account the Heckel plot region of plastic deformation, acceptable hardness/tensile strength (manufacturability and tabletability profiles), friability results, and disintegration time (4.0 ± 0.5 min), the tablets prepared at compression force of 9 kN (compaction pressure of 316.7 MPa) were used for the dissolution testing. In the dissolution media with a pH of 1.2, 4.5, and 6.8, test tablets displayed a dissolution rate higher than 85% per 15 min (Figure 9) and thus met the proposed biowaiver requirements [2].

## 5. Conclusions

This feasibility study demonstrated a successful attempt to produce high (90.9%, *w*/*w*) drug-loaded 100 mg dose immediate-release tablets of isoniazid utilising a widely used, common set of excipients (namely: Kollidon^®^ 25, Polyplasdone^TM^ XL-10, Resistamyl^®^ 347, and magnesium stearate), milling of raw substance, fluid-bed wet granulation with 15% (*w*/*w*) aqueous solution of Kollidon^®^ 25, and industrial-scale high-speed tableting with tablet press Korsch XL 100 (80 rpm; 62,400 tab/h). Obtained granulates had good mass flow rates (5.5 ± 0.3 g/min) and Hausner ratio (1.09) at given *D*_50_ particle size of 137.5 µm and have satisfied the tableting requirements. The high-speed tableting process at different tableting compaction force was in the range of 4.8–15.5 kN and was illustrated with the Heckel plot, manufacturability, tabletability, compactability, and compressibility profiles as well as supported with ejection force and tablet removal force data. The unchanged solid state after the manufacturing process of isoniazid was confirmed by DSC. Obtained results allowed for deeper understanding of the tableting process and data driven choice of compaction force. The tablets obtained at compaction force of 9.0 kN demonstrated an acceptable disintegration time of 4.0 ± 0.5 min and a dissolution rate higher than 85% per 15 min which meets the biowaiver requirements.

## Figures and Tables

**Table 1:** Granulate and tablet composition.

	Per Granulation		Per 1 Tablet		True Density
Ingredients	g	% (*w*/*w*)	mg	% (*w*/*w*)	mg/mm^3^
Isoniazid	2000.0	96.9	100.0	90.9	1.420
Kollidon^®^ 25	65.0	3.1	3.3	3.0	1.180
Resistamyl^®^ 347	–	–	3.7	3.3	1.516
Poliplasdone^TM^ XL-10	–	–	2.5	2.3	1.220
Magnesium stearate	–	–	0.6	0.5	1.096
Deionised water	368.0	NA	–	–	NA
**∑**	**2065.0**	**100.0**	**110.0**	**100.0**	**1.410 ^Th^**

**^Th^**—theoretically calculated true density of composition.

**Table 2:** Melting point and melting endotherm of raw substance and crushed tablets (*n* = 3).

	Raw Substance		Crushed Tablets	
	Av.	S.D.	Av.	S.D.
**Melting point, °C**	178.1	0.3	178.3	1.6
**Melting endotherm, J/g**	228.6	2.2	204.3	5.6

**Table 3:** Tableting characteristics: measured and calculated.

**Set**	Compaction Force, kN		**15.5**	**14.0**	**11.3**	**9.9**	**9.0**	**8.0**	**6.0**	**4.8**
**Measured**	Tablet hardness, N	Av.	87	91	89	83	78	73	65	60
		S.D.	8.6	5.1	3.9	2.5	4.3	6.3	7.1	6.9
	Tablet weight, mg	Av.	111.0	113.0	113.5	112.8	112.9	113.2	111.5	112.0
		S.D.	1.7 < S.D. < 5.5							
	Tablet thickness, mm	Av.	2.90	2.97	3.00	3.04	3.10	3.21	3.29	3.42
		S.D.	0.01							
**Calculated**	Compaction pressure, MPa		548.5	495.4	398.1	350.3	316.7	283.1	210.5	169.9
	Tensile Strength, MPa	Av.	3.18	3.25	3.15	2.90	2.67	2.42	2.09	1.86
		S.D.	0.32	0.18	0.14	0.09	0.15	0.21	0.23	0.21
	Solid fraction	Av.	0.961	0.955	0.950	0.931	0.914	0.888	0.848	0.822
		S.D.	0.008	0.006	0.006	0.007	0.004	0.003	0.002	0.002

## Data Availability

The data presented in this study are available on request from the corresponding author.
